# Exploring links between vitamin D deficiency and COVID-19

**DOI:** 10.1371/journal.ppat.1008874

**Published:** 2020-09-18

**Authors:** Mradul Mohan, Jerin Jose Cherian, Amit Sharma

**Affiliations:** 1 Parasite-Host Biology Group, National Institute of Malaria Research, New Delhi, India; 2 Division of Basic Medical Sciences, Indian Council of Medical Research, New Delhi, India; 3 Structural Parasitology Group, International Centre for Genetic Engineering and Biotechnology, New Delhi, India; Vallabhbhai Patel Chest Institute, INDIA

## Abstract

Coronavirus Disease 2019 (COVID-19) pandemic remains a major public health threat in most countries. The causative severe acute respiratory syndrome coronavirus 2 (SARS-CoV-2) virus can lead to acute respiratory distress syndrome and result in mortality in COVID-19 patients. Vitamin D is an immunomodulator hormone with established effectiveness against various upper respiratory infections. Vitamin D can stall hyper-inflammatory responses and expedite healing process of the affected areas, primarily in the lung tissue. Thus, there are ecological and mechanistic reasons to promote exploration of vitamin D action in COVID-19 patients. As no curative drugs are available currently for COVID-19, we feel that the potential of vitamin D to alter the course of disease severity needs to be investigated. Clinical studies may be undertaken to address the value of vitamin D supplementation in deficient, high-risk COVID-19 patients.

## SARS-CoV-2 infection and the cytokine storm

There are two critical questions that emanate from the title of this article. The first one is whether there is an association between vitamin D deficiency and susceptibility to Coronavirus Disease 2019 (COVID-19). The second is whether vitamin D administration to deficient individuals can prevent infection or alter the course of disease severity. Here, we have collated available evidence that addresses both these aspects of vitamin D in relation to COVID-19.

**Fig 1:**
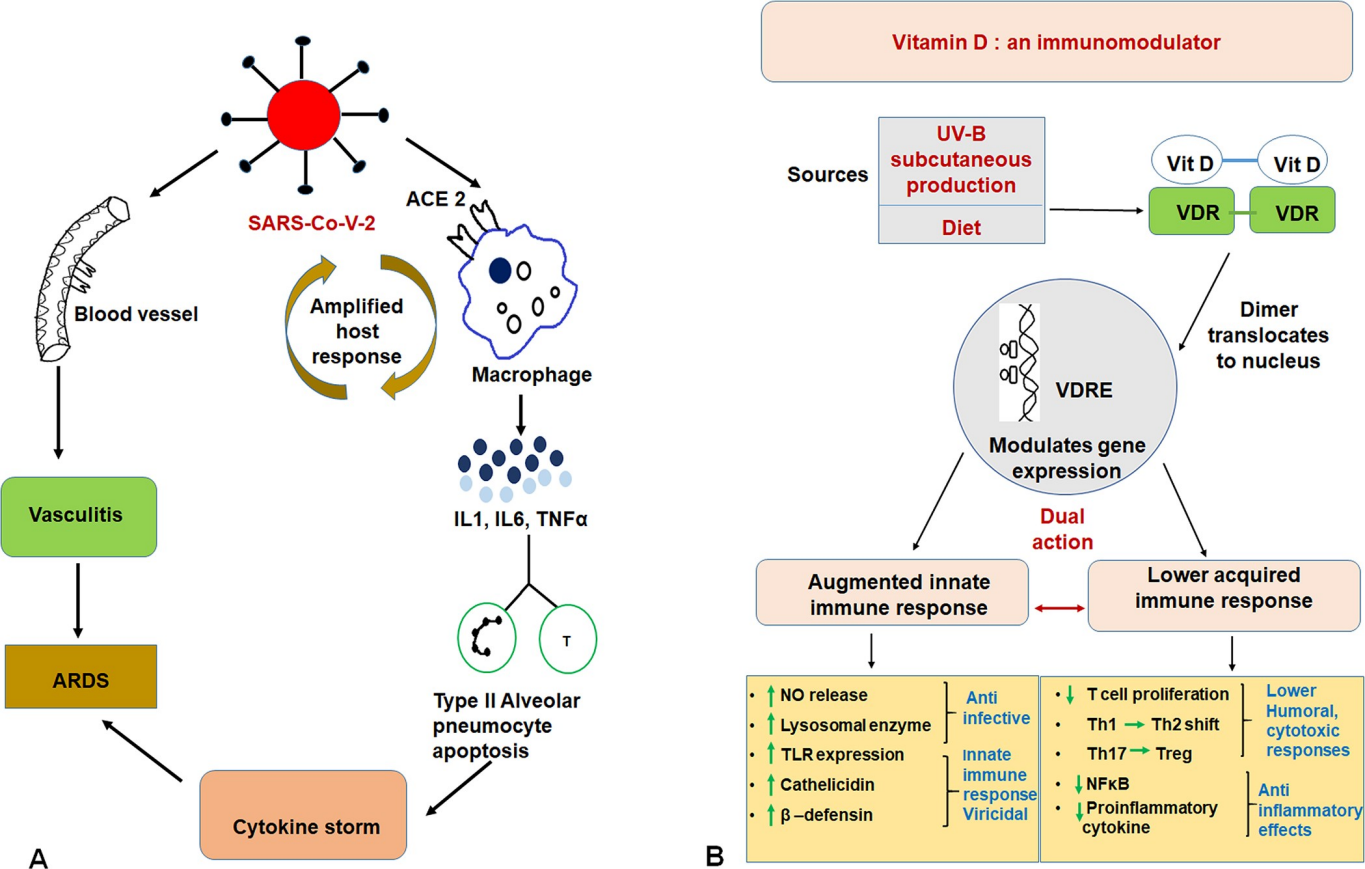
Fig 1A depicts the basic pathophysiology of COVID-19 and the development of ARDS. Fig 1B shows vitamin D’s dual action on immune response and inflammation. Vitamin D is capable of modulating the expression of various genes which results in augmented innate immune response and lower acquired immune response. ACE 2, angiotensin-converting enzyme 2; ARDS, acute respiratory distress syndrome; COVID-19, Coronavirus Disease 2019; IL, interleukin; NFκB, nuclear factor kappa-light-chain-enhancer of activated B cells; NO, nitric oxide; SARS-CoV-2, severe acute respiratory syndrome coronavirus 2; TH1, T helper type 1; TH2, T helper type 2; TH17, T helper type 17; TLR, toll-like receptor; TNFα, tumor necrosis factor alpha; TREG, regulatory T cell; UVB, ultraviolet B rays; VDR, vitamin D receptor; VDRE, vitamin D receptor element.

The severe acute respiratory syndrome coronavirus 2 (SARS-CoV-2) infects pulmonary epithelial cells using the angiotensin converting enzyme-2 (ACE-2) receptor [1]. Besides pulmonary epithelial damage, SARS-CoV-2 also infects macrophages through ACE-2 receptors and activates them [2]. Macrophages, neutrophils, and T cells get activated through sustained elevation of cytokines including interleukin (IL)-1, IL-6, and tumor necrosis factor (TNF) alpha, resulting in type 2 pneumocyte apoptosis, and in some patients a path that leads to acute respiratory distress syndrome (ARDS) [2]. The host responses are sometimes amplified by an overwhelming expression of pro-inflammatory cytokines [3]. This ‘cytokine storm’ is responsible for some of the serious manifestations of COVID-19 such as ARDS (Fig 1A) [3]. Hypoxemia and bilateral lung infiltration are features reminiscent of severe viral pneumonia that result from endothelial injury, excessive cytokines, and immune overkill [3,4].

## Vitamin D and the host immune system

Vitamin D can be classified as a steroid hormone and is produced in human skin from 7-dehydrocholesterol due to exposure to ultraviolet B rays (UVB; 280–315 nm range) from sunlight [5]. The subcutaneous production by UVB exposure is the principle source of vitamin D, whilst dietary sources include dairy products or fish liver oil (Fig 1A) [6]. Melanin is an indole-containing polymer produced in melanocytes which offer epidermis and hair their pigmentation. Melanin reduces the penetration of UVB, resulting in decreased vitamin D production in the skin [5]. For established genetic and environmental reasons, melanin expression in some ethnic groups like whites is reduced, and this has been linked to population-wide differences in vitamin D synthesis despite exposure to UVB [7].

Inside the cell, vitamin D binds to nuclear vitamin D receptors (VDRs) and the subsequently activated VDRs dimerise with themselves or with retinoid X receptors (RXR) and translocate to the nucleus to engage the vitamin D receptor element (VDRE) (Fig 1B). The VRDE regulates the expression of a numerous host genes like beta defensin and cathelicidin [8]. In addition, vitamin D levels can influence the expression of toll-like receptors that pivot the innate immune response as they recognise pathogenic proteins [9]. Other important genes regulated by vitamin D include beta defensins that can directly cleave the membrane of a virus and cathelicidins that are involved in the activation of macrophages, dendritic cells, and neutrophils [10]. For example, activated VDR can bind to the VDRE of the cathelicidin gene promoter and can lead to initiation of host defense against some viral infections [10]. Vitamin D also influences the innate immune system via expression of lysosomal enzymes and the release of nitric oxide wherein both contribute towards combating infection (Fig 1B) [11].

Vitamin D also plays an immune regulatory role via suppression of the adaptive immune responses in respiratory epithelial cells during viral infections [5,10]. This is manifested predominantly via dampening T cell proliferation and the resultant shift from T helper type 1 (Th1) cells to T helper type 2 (Th2) [12]. A reduced Th1 proliferation can be argued to result in lower levels of pro-inflammatory cytokines and diminished acquired immune responses, and these may be counterproductive in mounting a successful immune response against a virus (Fig 1B). Vitamin D also influences T cell maturation and can divert the development of inflammatory T helper type 17 (Th17) cell mass towards anti-inflammatory regulatory T cell (T-reg cell) populations [5,12]. In this manner, vitamin D can reduce the ‘in milieu’ levels of pro-inflammatory cytokines including IL-1, IL-6, IL-12, TNF alpha, and IL-17 whilst augmenting the anti-inflammatory IL-10 [5,10]. Reduced expression of pro-inflammatory cytokines restrains the differentiation and activation of various immune cell types and can prevent immune-mediated injury [5]. Additionally, vitamin D directly inhibits the nuclear factor kappa-light-chain-enhancer of activated B cells (NFκB) pathway, thus reducing the expression of pro-inflammatory cytokines [13]. Hence, via its opposing actions on cytokine regulation and T cell differentiation, vitamin D plays a complex dual role in immunopathology (Fig 1B) [5,10]. Some authors have also postulated that vitamin D may down-regulate ACE-2 receptors and thus can have protective effects in COVID-19 [14].

## Vitamin D deficiency and COVID-19

Lips and colleagues have recently evaluated the mean levels of vitamin D in populations across approximately 40 countries and have shown >50% deficiency, especially amongst the care home residents (mostly the elderly) [15]. As the COVID-19 pandemic count continues to rise in many countries including India, it is noteworthy that a substantial population of India (approximately >70%) are vitamin D deficient (<20 ng/ml) [16]. This deficit amongst urban India may be attributed to avoidance of sunlight and dietary deficiency. Poor diet may also be one of the factors for low vitamin D levels in the Indian population [16]. The situation is no better in rural India where despite plenty of opportunities for exposure to sunlight, the populations nonetheless show prevalence of vitamin D deficiency, possibly attributable to various factors including content of phytates/phosphates in the Indian diet [16]. Equally, in Europe, approximately 40% of the population is deficient (<20 ng/ml), but in its darker-skinned citizenry the prevalence is higher compared to whites [17,18]. Similarly, approximately 24% of U.S. citizens and approximately 37% of Canadians are deficient in vitamin D, but the problem is mostly limited to their nonwhite communities [18]. Vitamin D deficiency may additionally stem from various socioeconomic differences such as sun exposure time (occupation), sun exposure surface area (clothing and other customs), and diet, and thus these factors are additional confounders [19].

Studies have both recognised [20] and rejected [21] the association between vitamin D deficiency and viral respiratory infections. The differences in study methodology, demographics, vitamin D levels, VDR mutations, and supplement dosages have been targeted for their nonconforming nature of evidence [22]. Meanwhile, a recent systematic review and meta-analysis has concluded that vitamin D has potential in preventing respiratory infections, especially in those who have high levels of deficiency [23]. Whilst numerous factors interplay in determining the outcome of COVID-19 patients, ecological correlations of vitamin D levels to disease incidence and mortality have shown some crude associations [17,24]. There could also be other indirect correlations between vitamin D deficiency and conditions such as diabetes and hypertension which in themselves associate with severity of COVID-19 [25].

A retrospective, multicentric study has suggested that whilst COVID-19 patients (who were deficient in vitamin D) generally had poor outcomes, those with high levels of vitamin D fared better outcomes [26]. A review published by Rhodes and colleagues has concluded that there was substantial ecological evidence to correlate vitamin D deficiency with severity of COVID-19 infection [27]. Jain and colleagues have noted that African Americans with vitamin D deficiencies as well as those with poorer COVID-19 outcomes may stand to benefit from supplementation [28]. Merzon and colleagues have made similar conclusions in an Israeli population [29].The above mentioned studies thus suggest that vitamin D supplementation may help patients with COVID-19, despite the web of confounders the recommendations tangle with [17,30].

Meanwhile, others have argued that the risk of severe COVID-19 infections in certain populations cannot be adequately explained by their vitamin D deficiency status [31]. Another study indicates that vitamin D supplementation reduces the Th2 responses during *Aspergillus* infection amongst patients of cystic fibrosis [32], and it is noteworthy that pulmonary aspergillosis is a secondary infection amongst COVID-19 critical patients [33]. Therefore, vitamin D supplementation could possibly make patients with COVID-19 prone to secondary infection with *Aspergillus* spp. even though such associations have not been documented yet. Thus, the yin-yang of vitamin D supplementation during the COVID-19 era continues.

## Conclusions

Immune dysregulation is a key feature of severe COVID-19. Therefore, the restoration of immune balance to prevent the hyper-inflammatory cytokine storm is a reasonable strategy to combat disease severity in COVID-19. However, conventional immunomodulator therapies may be a double-edged sword as they can unintentionally suppress protective immune responses. In this context, vitamin D’s dual roles of initially controlling viral replication and later dampening the hyper-inflammation are tantalising. This is consistent with the observation that low levels of vitamin D (or indeed polymorphisms in the VDR) may adversely impact the outcome of patients with COVID-19 [17,24–30].

Given that vitamin D has shown benefits in certain viral respiratory infections including in COVID-19, the roles of this hormone warrant further exploration. During the COVID-19 pandemic, an estimation of 25-hydroxyvitamin D can be done in the high-risk, comorbid, and elderly populations who are SARS-CoV-2 positive. Vitamin D supplementation may be considered in such risk groups given that it is relatively cheap and safe and is widely available as a supplement. However, caution is vital here as there is a need for robust evidence to substantiate vitamin D’s routine clinical use in COVID-19. Similarly, clinical guidance will be critical in preventing vitamin D toxicity arising from irrational supplementation or in people who have polymorphisms in their cytochrome P450 family 24 subfamily A member 1 (CYP24A1) and/or VDRs [34]. Some commonly used medicines such as statins, antituberculars, and antiepileptics are known to interact with vitamin D, and thus clinical supervision is mandated in these cases [35]. Vitamin D toxicity, although rare, may manifest as dehydration and result in high calcium uptake associated with vitamin D supplementation for prolonged periods.

The authors here do not endorse the use of vitamin D for treatment or prevention of COVID-19 infections but instead stress the need for more robust research that can address the early correlations noted above. We would like to encourage research that helps answer some of the critical questions raised in this paper. Our hypothesis could be explored by a case control study (such as the COVIDENCE UK study) to observe the frequency of vitamin D deficiency amongst patients with poor COVID-19 outcomes. Case reports on the compassionate use of vitamin D may also help probe vitamin D’s therapeutic effects. Randomised controlled trials such as those initiated in Spain (NCT04334005), Argentina (NCT04411446), France (NCT04344041), and Iran (IRCT20200324046850N1) would bring forth superior evidence to link healthy levels of vitamin D with better outcomes in COVID-19.
