# Correction: Exploring links between vitamin D deficiency and COVID-19

**DOI:** 10.1371/journal.ppat.1010985

**Published:** 2022-11-21

**Authors:** Mradul Mohan, Jerin Jose Cherian, Amit Sharma

The ORCID iD for the corresponding author is incorrect. Amit Sharma’s ORCID iD is: 0000-0002-3305-0034 (https://orcid.org/0000-0002-3305-0034).

The ORCID iD is missing for the first author. Mradul Mohan’s ORCID iD is: 0000-0002-5164-9921 (https://orcid.org/0000-0002-5164-9921).
